# Knowledge and Attitude Related to Sexually Transmitted Infections and Contraceptive Use Among Patients Attending the Venereology Outpatient Department in Thiruvallur: A Cross-Sectional Study

**DOI:** 10.7759/cureus.76281

**Published:** 2024-12-23

**Authors:** Sonia S, Vengatachalapathy P, Arthipriya Kumaravel, Jannath Hameeda Banu M, Adikrishnan S

**Affiliations:** 1 Dermatology, Venereology, Leprosy, Sri Ramachandra Institute of Higher Education and Research, Chennai, IND; 2 Dermatology, Venereology, Leprosy, Government Medical College and Hospital, Thiruvallur, Chennai, IND; 3 Community Medicine, Stanley Medical College, Chennai, IND

**Keywords:** attitude, awareness, contraceptive, education, homosexuals, knowledge, questionnaire, sexual risk behaviour, sti, venereology

## Abstract

Introduction

Sexually transmitted infections (STIs), which contribute to a significant amount of mortality and morbidity in the reproductive life of adults, are infections that can be prevented by healthy sexual behavior and education about the same. This study aims to assess the knowledge and attitude toward STIs and contraceptive use among patients attending the Venereology Outpatient Department (OPD).

Methods

A hospital-based cross-sectional study was conducted among patients more than 18 years of age attending the Venereology OPD, Government Medical College and Hospital, Thiruvallur, India, from March to May 2024. The sample size was calculated to be 458.

Data were collected using a self-administered, semi-structured questionnaire divided into four sections: sociodemographic characteristics, sexual risk behavior and their awareness of contraceptive use, knowledge of STI symptoms and transmission, and attitude of the patients toward STIs. Ethical approval (IEC/4/2022 dated 08.02.2024) and participant consent were obtained. Statistical analysis, including the chi-square test, was performed and results were presented.

Results

Of the study population, 50.4% were males, 49.1% were females, and 0.4% (n=2) were transgender individuals. STI screening and treatment rates were 22.1% and 19.4%, respectively. Contraceptive use was highest among bisexual individuals (50%), followed by homosexual (15%) and heterosexual individuals (11.8%). Similarly, treatment for STIs was reported in 55% of homosexuals, 50% of bisexuals, and 15.8% of heterosexuals. Among participants with multiple sexual partners, 30% reported contraceptive use as compared to less than 10% of those with single partners. Treatment for STIs was also higher in participants with multiple partners (65.2%) as compared to those with single partners (11.5%). The median knowledge score about STIs was 6.32, with only 44.3% of participants demonstrating good knowledge. The median attitude score was 52.63, with 44.3% showing a positive attitude toward STIs.

Conclusion

The overall knowledge and attitude towards STIs were below par. Thus, the current study has found the need for sexual health education among patients and to create awareness of contraceptive use to fill gaps in existing knowledge, which can further reduce the risk of STIs among them.

## Introduction

The term “sexually transmitted infection” (STI) refers to a pathogen that causes infection through sexual contact, whereas the term “sexually transmitted disease” (STD) refers to a recognizable disease state that has developed from an infection. STIs can be caused by bacteria, viruses, protozoa, fungi, and ectoparasites. These agents can spread through skin-to-skin contact, infected needles, vaginal, oral, and anal intercourse, as well as mother-to-child transmission during pregnancy and lactation. STIs contribute to a significant amount of maternal morbidity, ectopic pregnancy, infant illness and death, malignancy, infertility, urethritis, epididymitis, prostatitis, and increased susceptibility to HIV [1].

In 2020, WHO estimated 374 million new infections with one of four STIs: chlamydia (129 million), gonorrhea (82 million), syphilis, and trichomoniasis (156 million) [2]. According to projections, the annual incidence of RTIs and sexually transmitted infections in India is 5%, or about 40 million per year [3]. Studies suggest that 6% of the adult population in India is infected with one or more STIs or reproductive tract infections (RTIs) [4].

In India, according to the National Family Health Survey (NFHS-5), it was also noted that the STIs among the age group of 15-24 years were 5.6% in males and 2.0% in females [5]. A positive, respectful approach to sexuality and relationships, with the possibility of having pleasurable and safe sexual experiences free of coercion, discrimination, and violence, is essential for good sexual health [6].

In addition, the general public engages in riskier sexual practices and premarital sex more frequently because they are less aware of STIs, their symptoms, and their causes. To increase awareness among them and develop interventions to overcome barriers, it is crucial to understand the region-specific factors influencing knowledge and attitude concerning STI and contraceptive use. There are only a few studies on knowledge assessment, attitudes toward STIs, and contraception use [7].

Thus, identification of the gaps in the current knowledge regarding the use of contraceptives and sexual health education for patients will possibly help the medical fraternity to lower the risk of STIs among patients and ultimately contribute to the achievement of sustainable development goals, which include good health and well-being. By assessing this, the unmet need for contraceptive use would be addressed also. Thus this study aims to measure the knowledge and attitude toward STIs and contraceptive use among patients attending the Venereology OPD.

Objectives

The objective of the current study was to assess the knowledge and attitude about STI among the study participants attending the Venereology OPD. The study also assessed their sexual risk behavior and contraceptive use. The factors influencing the knowledge and attitude about STI and contraceptive use were also assessed in the study.

## Materials and methods

This is a hospital-based cross-sectional study conducted among all patients of all genders, who are over 18 years old, attending the Venereology OPD, Government Medical College Hospital, Thiruvallur, India, from 01 March 2024 to 31 May 2024. The study was conducted with ethical clearance from the Institutional Ethics Committee of Government Medical College, Thiruvallur (IEC/4/2022 dated 08.02.2024). The inclusion criteria included all patients more than 18 years of age attending the Venereology OPD and willing to give written consent to participate in the study. The exclusion criteria were a history of neurological or psychiatric conditions that can impair judgment to questions asked. Purposive sampling was used to recruit the study participants.

The sample size was calculated according to the following study by Dorji et al. in Bhutan where the proportion of university students with good knowledge about STI was 53.2% [8]. With this, the sample size was calculated to be 458 using the formula {Zα2×pq} ÷ d2, and assuming a confidence interval of 95% and an absolute precision of 5%. Expecting a non-response rate of 20%, the final sample size was arrived at (382 + 76 = 458) [9].

Data were collected using a self-administered, semi-structured questionnaire divided into four sections (Appendices), namely, the socio-demographic characteristics of patients (including their age, gender, marital status, residence, stay, occupation, education, religion, and the number of children), sexual risk behavior of the patients and their awareness on contraceptive use (history of sexual activity, mode of contact, age at first sexual activity, contraceptive use, history of STI screening and treatment, sexual activity under the influence of alcohol/drug use, adequacy of sexual health information, discussion about contraceptives with health personnel, easy availability of contraceptives), knowledge of STI, and attitude towards STI. The third part had 12 questions with multiple correct answers designed to test knowledge of STI identification, transmission, symptoms, and risk factors. A score of "1" was offered for correct answers and "0" for incorrect answers or who answered 'don't know.' The total knowledge score for all the correct answers was calculated. Using the mean score as the cutoff, the participants were grouped as subjects with good and poor knowledge. Similarly, the fourth part of the questionnaire measured the attitude towards STI through 15 questions, and the answers were recorded on a 5-point Likert scale of strongly agree, agree, neutral, disagree, and strongly disagree. Those with a good attitude received a score of "1" while poor attitude received a score of "0." With the mean score as the cutoff, the participants were grouped into good and poor attitudes.

Participants were interviewed in private consultation rooms to ensure confidentiality. The questions were clearly explained to the patients in their local language or a language they found comfortable. Then, the patients were asked to answer the questions, and the answers were documented by the interviewer. The responses of the subjects were kept anonymous. The collected data were entered in Microsoft Excel version 11 (Microsoft Corporation, Redmond, WA, US) and data validation was done. Statistical analysis was done using Statistical Package for Social Sciences (SPSS, version 16; SPSS Inc., Chicago, Il, US). The results are expressed in tables and graphs as necessary. Proportions of central tendency like mean and median are used to express the results. Comparative analyses were done using chi-square tests.

## Results

Socio-demographic characteristics of the patients

The age of the study participants was skewed (Kolmogorov-Smirnov test statistic: 0.086, p-value <0.001) in distribution, and the median age was noted to be 35 years (interquartile range: 26-46 years) while the mean age of the participants was 37.85 years (36.56-39.10) and the standard deviation was 13.83 years (12.98-14.61). Males and females (50.4% vs. 49.1%) were equally distributed among the study participants, with only two (0.4%) transgender patients, as shown in Table 1. The majority of the study participants were married. Also, more than half were from rural areas, and the majority (88.6%) were residing with their family members. Regarding the educational qualification of the study participants, the majority had completed higher secondary school (31.2%), followed by graduates (22.7%). Less than 10% were illiterate. Among occupations, the majority were homemakers (27.1%), followed by business people or those with private jobs (19.7%). Only two were commercial sex workers (0.4%) while around 15% did not specify their occupation. The majority were of the Hindu religion (81.4%). While 27.3% were unmarried, 35.4% had no children. The majority had two children (41.7%), as shown in Table 1.

**Table 1:** Sociodemographic characteristics of the study population

Sl. No.	Sociodemographic Characteristics		Number	Percentage
1	Sex	Male	231	50.4
		Female	225	49.1
		Transgender	2	0.4
2	Marital status	Married	319	69.7
		Unmarried	125	27.3
		Divorced/Separated	12	2.6
		Widowed	2	0.4
3	Residence	Rural	257	56.1
		Semi-urban	57	12.4
		Urban	144	31.4
4	Staying with families	Yes	406	88.6
		Away	52	11.4
5	Education	Illiterate	37	8.1
		Primary school	43	9.4
		Middle school	66	14.4
		Secondary school	42	9.2
		Higher Secondary	143	31.2
		Graduate	104	22.7
		Post-graduate	23	5.0
6	Occupation	Housewife	124	27.1
		Business/Private	90	19.7
		Labourer	60	13.1
		Student	48	10.5
		Farmer	34	7.4
		Driver	24	5.2
		Government job	7	1.5
		Commercial sex worker	2	0.4
		Others (Unspecified)	69	15.1
7	Religion	Hindu	373	81.4
		Christian	70	15.3
		Muslim	14	3.3
8	Number of children	Nil	162	35.4
		One	60	13.1
		Two	191	41.7
		Three or more	45	9.8

Sexual risk behavior of patients and their awareness of contraceptive use

Regarding sexual behavior, 370 of the participants (80.8%) responded they have had lifetime sexual relations. Among the females, 83.1% (n = 187/225) had sexual relations in their lifetime while 78.8% (n = 182/231) of the males have had sexual encounters previously. One of the two transgender patients has had lifetime sexual relations. Males and females were statistically comparable in terms of lifetime sexual relations (chi-square statistic: 1.362, p = 0.243). Among those who were sexually active, 2.8% reported that they were under the age of 18 when they had their first sexual experience. Less than 10%, i.e., 8.37% (n = 31/370), have mentioned they have paid for sex. Less than 1% (n = 4/370) reported that they have had sex with a commercial sex worker. About one in five (18.9%, n = 70/370) have had sex under the influence of alcohol, and one-fifth (19.4%, n = 72/370) have previously received treatment for sexually transmitted infections.

Among participants who experienced sexual interactions, the majority were heterosexual (94.06%, n = 348/370), followed by homosexual (5.42%, n = 20/370), and bisexual (0.52%, n = 2/370). Only 12.7% (n = 47/370) reported having multiple partners. Nine out of 10 (n = 333/370) had contact with a known person, 2.7% (n = 10/370) had contact with an unknown person, and 7.29% (n = 27/370) had contact with both. Regarding protection through sexual encounters, only 12.1% (n = 45/370) have had protected sex, whereas 81.08% (n = 300/370) had unprotected sex and 6.4% (n = 24/370) had both. 

The comparison of the proportion of subjects who had protected sex among various genders is described in Table 2, which shows that the proportion of males having protected sex was significantly higher than the proportion of females having protected sex, and the difference was statistically higher. It was also observed that the proportions of subjects having sex with multiple partners (males vs. females: 14.8% vs. 9.6%, chi-square statistic: 10.283, p = 0.036) and unknown persons (males vs. females: 4.9% vs. 0.5%, chi-square statistic: 21.551, p = 0.001) were higher among males than females.

**Table 2:** Comparison of sexual behaviors and risks based on genders, sexual orientation, and number of partners #: chi-square test statistic, *: p-value <0.05 is considered significant, n: number, %: percentage

Sl. No.	Characteristics		Protected Sex n (%)	Screened for STI n (%)	Treated for STI n (%)
1	Gender	Male (n=182)	31 (17.03)	44 (24.18)	34 (18.68)
		Female (n=187)	14 (7.49)	55 (29.14)	37 (19.79)
		Transgender (n=1)	0 (0)	1 (100)	1 (100)
		Test statistic (p-value)	7.818^#^ (0.005*)	1.156^#^ (0.282)	0.073^# ^(0.787)
2	Sexual orientation	Heterosexual (n=348)	41 (11.8)	75 (21.6)	55 (15.8)
		Homosexual (n=20)	3 (15)	13 (65)	11 (55)
		Bisexual (n=2)	1 (50)	1 (50)	1 (50)
		Test statistic (p-value)	0.183^#^ (0.667)	19.501^#^ (<0.0001*)	19.696^#^ (<0.0001*)
3	Sexual partner	Single (n=323)	31 (9.6)	56 (17.3)	37 (11.5)
		Multiple (n=46)	14 (30.4)	33 (71.7)	30 (65.2)
		Test statistic (p-value)	16.227^#^ (0.0001*)	65.001^#^ (<0.0001*)	77.801^#^ (<0.0001*)

A comparison of STI screening and treatment among various genders is described in Table 2, which depicts that the rates of STI screening were higher among females than males (24.18% vs. 29.14%), but the difference was not statistically significant. Both the transgender patients, the one who had lifetime sexual relations and the one who had never had sex, had undergone screening for STIs. Similarly, the proportion of females having been treated for STI was higher, but not statistically, than the males (18.68% vs. 19.79%).

A comparison of the proportion of subjects who had protected sex, STI screening, and treatment among various sexual orientations, as stated in Table 2, shows that both heterosexual and homosexual participants had a similar proportion of people having protected sex. While the screening for STIs was higher among the homosexual and bisexual participants than the heterosexual patients, the proportion of people who underwent STI treatment was also higher among homosexual and bisexual participants. The difference was statistically significant in both instances.

Table 2 also compares protected sex, STI screening, and treatment based on the number of partners, which shows that protected sex among those with single partners is lower than among those with multiple partners. Participants with single partners and multiple partners undergo STI screening at rates of 17.3% and 71.7%, respectively. This was statistically higher among the latter group, and STI treatment rates among the two groups are statistically different, with rates higher among those with multiple partners.

Among the 370 subjects who have had lifetime sexual relations, 36.75% had used some form of contraception while 8.9% (n = 33) had used condoms, 7% (n = 26) had used permanent sterilization methods, 5.6% (n = 21) had used intrauterine contraceptive devices, 3.2% (n = 12) had used oral contraceptive pills (including emergency pills), 1.6% (n = 6) had followed the calendar method, 1.3% (n = 5) had used the withdrawal method, and 0.5% (n = 2) had used injectable contraceptives, of which only the first method is protective against STIs. The reasons given for not using a condom and other contraceptives were demonstrated in Table 3, where it was observed that unawareness was the most common cause for both methods, followed by embarrassment related to condoms and fear of the side effects of other contraceptive methods.

**Table 3:** Reasons for not using different methods of contraception OCP: oral contraceptive pills; n: number, %: percentage

Reasons/Methods	Condom: n (%)	OCPs and other methods: n (%)
Do you feel ashamed to ask?	56 (15.1)	35 (9.4)
Social humiliation	41 (11.08)	35 (9.4)
Fear of harmful effects	-	70 (18.9)
Less pleasure during sex	49 (13.24)	-
Unwillingness of partner	31 (8.3)	-
Not readily available	16 (4.3)	24 (6.4)
Discardal issues	14 (3.7)	-
Unaware	103 (27.23)	115 (31.08)
Other reasons - Felt no need	4 (1)	3 (0.8)
Other reasons - Planning for pregnancy	5 (1.3)	1 (0.2)

Among the study participants, 22.1% (n = 101/458) had undergone STI screening while 28.4% (n = 130/458) had accessed sex education. In terms of sources of information on sex education, electronic media and the Internet were the most-mentioned sources, as seen in Table 4. Less than one-third (31.2%, n = 143/458) had ever discussed contraception with their healthcare providers. Barrier contraceptives were easily available to 29.9% (n = 137/458) of the participants as per their statements.

**Table 4:** Various sources of information on sex education n: number, %: percentage

Sl. No.	Source of information on sex education	n (%)
1	Electronic media	159 (34.7)
2	Internet	147 (32.1)
3	Friends or relatives	96 (21)
4	Hospitals or health care workers	96 (21)
5	Print media	71 (15.5)
6	Public talks or seminars	64 (14)
7	Teachers or schools	63 (13.8)
8	Posters	46 (10)

Patients' knowledge of STI symptoms and transmission

**Figure 1:**
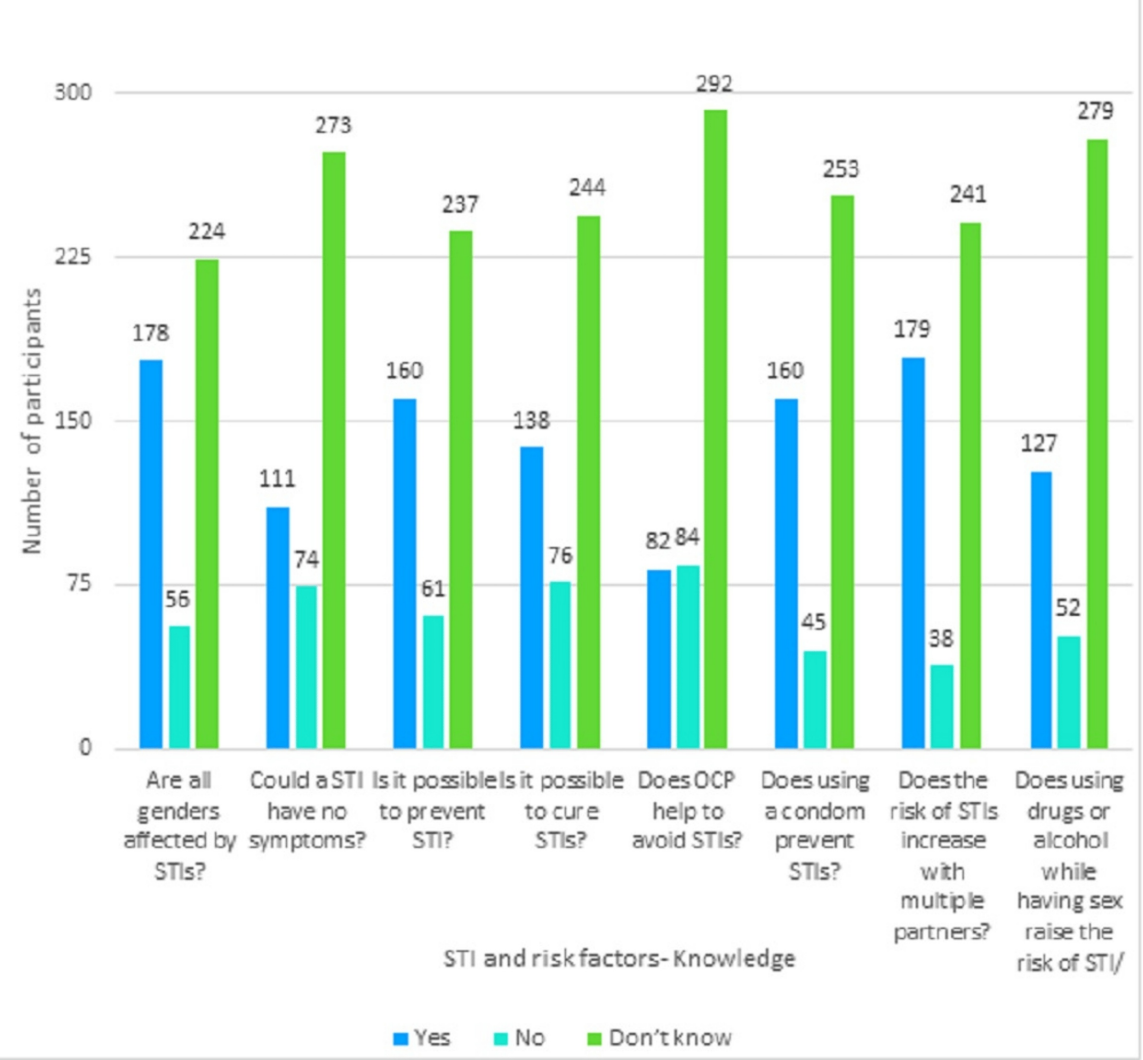
Knowledge regarding STIs and their risk factors STI: sexually transmitted infections

The mean knowledge score on STI is 6.32 (range 5.75-6.89). As the score was non-normally distributed (Kolmogorov-Smirnov test statistic: 0.131, p-value<0.001), the median value was used as the cut-off. Forty-four point three percent (44.3%; n = 203) of the participants had scored more than six (median value = 6) and were deemed to have good knowledge of STI. Figure 1 shows the proportion of participants giving the right answers to the questions regarding knowledge of STIs and their risk factors. While 35% identified condoms as a protective factor for STIs, 17.9% wrongly identified oral contraceptive pills as a preventive measure. Knowledge of risky behaviors like multiple partners and alcohol influence was present among 39.1% and 27.7%, respectively. More than two-thirds (38.9%) knew that STIs affect all genders. Around 35% knew that STIs can be prevented, another 35% knew that condoms prevent STIs, and 30.1% correctly said that STIs can be cured, as shown in Figure 1.

Human immunodeficiency virus (HIV)/acquired immunodeficiency syndrome (AIDS) was rightly identified as an STI by nearly half of the participants (49.1%), followed by syphilis (15.1%), while 39.7% (n = 182/458) could not correctly mention any of the STIs from the list provided in the questionnaire as shown in Table 5. Cervical cancer, foul-smelling vaginal discharge, abdominal pain, and genital ulcer were also mentioned as STIs by some participants though the number was less than 10 in each case.

**Table 5:** Common STIs identified by the study participants * Incorrectly identified as STIs STIs: sexually transmitted infections, HIV: human immunodeficiency virus, AIDS: acquired immunodeficiency syndrome

Identification of STIs	Number	Percentage
HIV/AIDS	225	49.1
Hepatitis B	42	9.2
Hepatitis C	57	12.4
Syphilis	69	15.1
Gonorrhea	44	9.6
Herpes	24	5.2
Tuberculosis*	23	5.0
Rabies*	14	3.1
Asthma*	14	3.1
Do not know	182	39.7
Others	7	1.4

**Figure 2:**
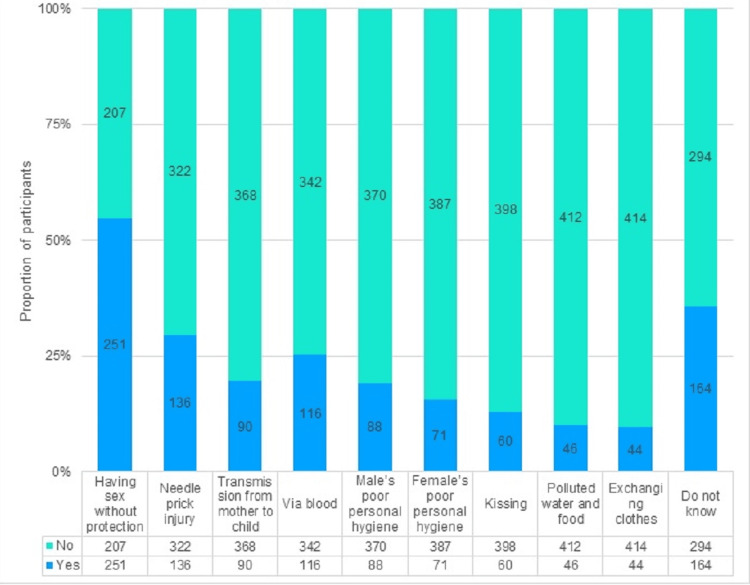
Knowledge of STI transmission STI: sexually transmitted infection

More than half mentioned unprotected sex (54.8%) as a mode of STI transmission. Thirty percent (30%) mentioned sharing contaminated needles, 25% reported that STIs can be transmitted through blood, and 20% correctly identified mother-to-child transmission. Less than 20% (19.2%) mentioned poor male hygiene as a transmission mode while 15.5% mentioned poor female hygiene. Around one-third (36%) were unaware of any of the STI transmission routes, as shown in Figure 2.

**Figure 3:**
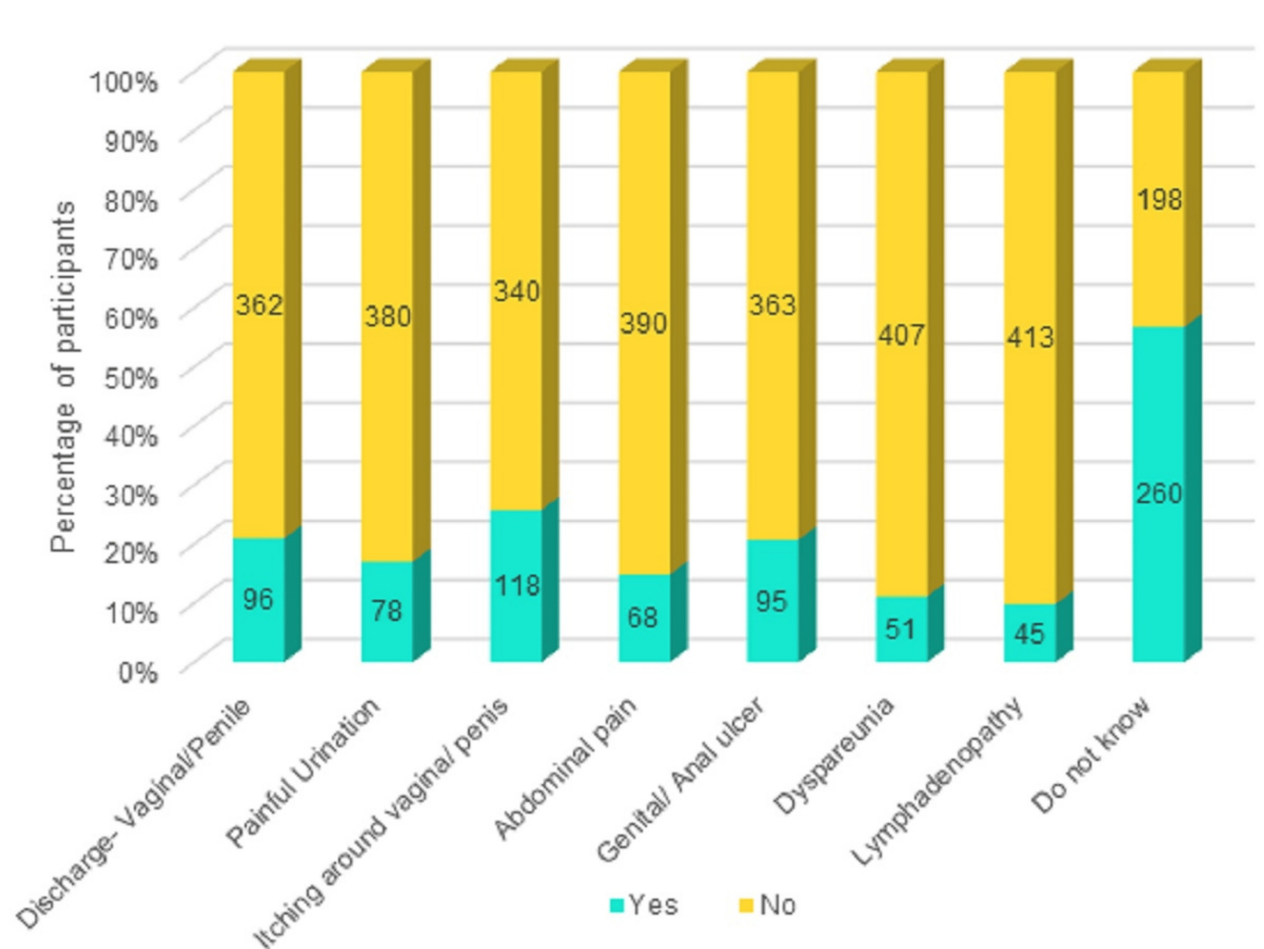
Knowledge about the symptoms of STIs STIs: sexually transmitted infections

Itching was the most-identified symptom by 25.8% of the participants, followed by genital discharge (21%), and genital or anal ulcer (20.7%). Most participants (56.8%) did not know any symptoms of STI, as shown in Figure 3.

The most common complication reported by the participants was cervical cancer (20.1%), and more than 60% (63.8%) were not aware of any complication of STIs, as shown in Table 6.

**Table 6:** Knowledge about the complications of STIs STIs: sexually transmitted infections, n: Number, %: percentage

Sl.No.	Complications of STIs	n (%)
1	Cervical cancer	92 (20.1)
2	Other organ involvement	61 (13.1)
3	Ectopic pregnancy	49 (10.7)
4	Infertility	48 (10.5)
5	Abortions	39 (8.5)
6	I don’t know	292 (63.8)

Attitude of patients toward STI

**Figure 4:**
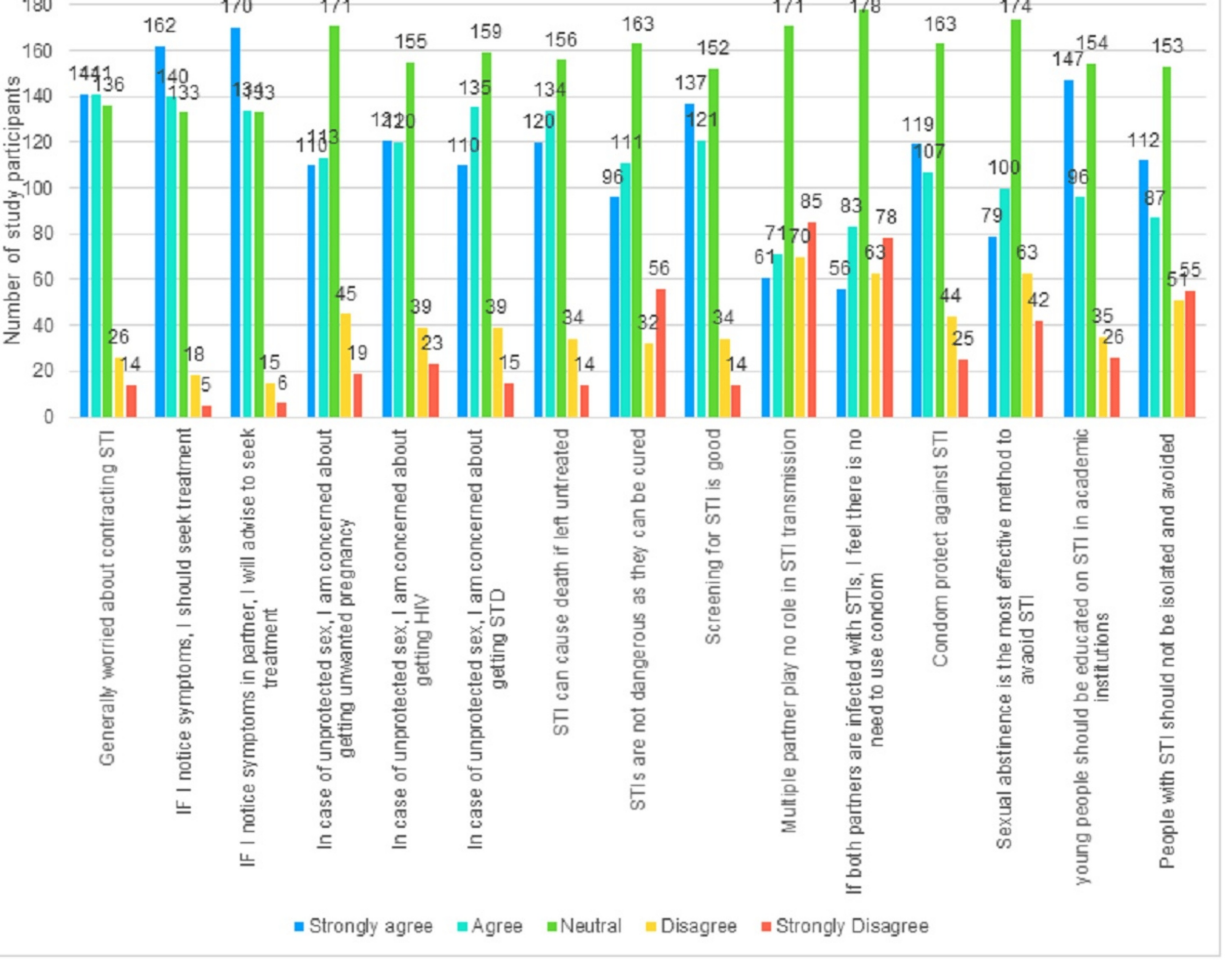
Attitude of the study participants regarding STIs STIs: sexually transmitted infections, STDs: sexually transmitted diseases

The mean attitude toward STI score was 52.63 (range: 51.88-53.32). As the attitude score was non-normally distributed (Kolmogorov-Smirnov test statistic: 0.154, p-value <0.001), the median value was used as a cut-off. Forty-four point three percent (44.3%; n = 203) of the participants who had scored more than 52 (median value=52) were deemed to have good knowledge of STIs. The attitude toward STI is shown in Figure 4. Almost 49.3% (n = 226/458) agreed that the use of condoms protects against STIs. Twenty-eight point eight percent (28.8%; n = 132/458) believed that having multiple partners did not contribute to the transmission of STIs. Thirty point three percent (30.3%; n = 139/458) said that there was no need to use a condom if both partners had STIs. Forty-five point one percent (45.1%; n = 207/458) considered STIs not dangerous, as they can be cured. Although 48.6% (n = 223/458) were concerned about unprotected intercourse leading to unwanted pregnancies, 53.4% (n = 245/458) were also concerned about getting an STI. Fifty-six point three percent (56.3%; n = 258/458) said that screening for STI is good. Sixty-five point nine percent (65.9%; n = 302/458) also said that they would seek treatment if they had symptoms of an STI.

## Discussion

The findings of this study provide valuable insight into the sexual risk behaviors and contraceptive use, knowledge, and attitudes toward STIs among patients attending the Venereology OPD at GMCH, Thiruvallur. One of the most notable findings is the high percentage of participants from rural regions (56%), which is significantly higher than the 22.2% reported in a study by Ali et al. [10]. This suggests that rural populations may have a higher prevalence of sexual health concerns, possibly due to limited access to education and healthcare services, poverty, and infrastructure deficits.

The low percentage (2.8%) of participants reporting their first sexual experience under the age of 18 is also noteworthy and much lower than the 13% reported by Muhammed et al. [11]. Cultural, social, or educational factors specific to the study population may influence this delayed sexual initiation. The prevalence of high-risk sexual behaviors in the current study shows both encouraging and concerning patterns. For instance, the low rate (less than 1%) of individuals reporting sex with commercial sex workers (CSWs) aligns closely with the findings of Decker et al. [12], suggesting that sex with CSWs is not a significant risk factor in this population. However, 8.37% of participants reported paying for sex, which, while lower than the 26.9% reported by Nicola Doring et al. [13], still indicates a potential area of concern for STI transmission. Additionally, 18.9% reported having sex under the influence of alcohol, a higher rate than the 16.7% reported by Ali et al. [10], suggesting that alcohol may contribute to riskier sexual behaviors in this population. Twelve point seven percent (12.7%) of participants reported having multiple sexual partners, a rate lower than the 16.19% observed in the study by Dorji T et al. [8]. The lower percentage could indicate either more conservative sexual behaviors in this population or better awareness of the risks associated with multiple sexual partners. Only 2.7% of participants reported contact with an unknown person, significantly less than the 13.6% observed by Maria et al. [14]. This suggests that anonymous or casual sexual encounters may be less common in the study population, which could potentially lower the risk of STI transmission. The high rate of unprotected sex (81.08%) observed in this study is concerning, especially when compared to the 40.9% reported by Dereje et al. [15]. The study found that just 7.49% of females practiced protected sex, a figure notably lower than the 50.6% reported by Dereje et al. [15]. This discrepancy may be attributed to societal pressures on women to bear children, as well as a lack of knowledge regarding barrier contraception and other contraceptive methods, fear of side effects, male partner dominance, or limited autonomy. Fifteen percent (15%) of homosexual participants engaged in protected sex, which is higher than the overall population but significantly lower than in the study by Milan et al. [16]. Despite the positive indication of awareness and preventive measures within this subgroup, the low percentage compared to other studies indicates the need for more efforts to promote consistent condom use in same-sex relationships. This could be due to barriers like social stigma or lack of access to LGBTQIA+ specific sexual health services, emphasizing the need for inclusive healthcare initiatives. The study found that only 36.75% of participants had used contraception, a much lower rate than the 97% reported by Singh R et al. [17]. This low contraceptive use, particularly among women, highlights a major gap in sexual health services, education, and access to contraceptives. The study found that only 8.9% of participants preferred condoms as their contraceptive method, which is much lower than the 25.3% reported by Singh R et al [17]. On the positive side, the low use could be an opportunity for targeted interventions promoting condom use, as they not only prevent unwanted pregnancies but also protect against STIs. However, such campaigns would need to address common barriers, like concerns about decreased pleasure during sex or inconvenience. Interestingly, the study also found that lack of awareness, rather than perceived loss of pleasure or fear of side effects, was the primary reason for not using condoms or oral contraceptive pills (OCPs), contrasting sharply with findings from Dorji T et al. [8]. This suggests that education and awareness campaigns tailored to this population could be a key intervention strategy.

Only 28.4% of participants reported having accessed sex education, significantly lower than the 77.7% observed by Dorji et al. [8]. The downside of this gap is that individuals may rely on unreliable sources of sexual health information, leading to misconceptions. On the other hand, this represents an opportunity to advocate for increased implementation of comprehensive sex education programs in schools and communities to bridge this knowledge gap. Twenty-two point one percent (22.1%) of participants had undergone STI screening, a rate much higher than the 9% reported by Dorji et al. [8]. This is a positive finding, as regular screening is essential for early detection and treatment of STIs, potentially reducing transmission rates. The majority of participants (34.7%) cited electronic media as their main source of sex education, unlike the study by Subbarao et al., where teachers were the primary source [18]. The advantage of electronic media is that it provides easily accessible information, especially for those who may not have formal sex education. The disadvantage is that it can spread misinformation, thus emphasizing the need for credible, culturally appropriate online resources. Therefore, a balanced approach would combine the use of reliable online resources with the delivery of formal sex education by trained educators. Twenty-nine point nine percent (29.9%) of participants reported that barrier contraceptives were easily available, a figure much lower than the 64.5% reported by Dorji et al. [8]. Limited availability of contraceptives could hinder safer sexual practices and contribute to higher rates of unintended pregnancies and STIs.

Only 44.3% of participants had good knowledge about STIs, a lower figure than the 53.2% reported by Dorji et al. [8]. Only 49.1% of participants identified HIV/AIDS as an STI, which is much lower than the 94.2% reported by Vasudev et al. [19]. This finding highlights the importance of HIV education campaigns and suggests that the successful implementation of such initiatives could significantly improve public health outcomes related to HIV prevention. A majority of participants (55%) were aware that unprotected sex can lead to STIs, although this is still lower than the 69.1% observed in the study by Subbarao et al. [18]. The low awareness of common STI symptoms, such as genital itching (26%), compared to the 77% reported by Ali I et al. [10], further indicates a need for enhanced education. Thirty-nine point one percent (39.1%) of participants acknowledged that having multiple sexual partners increases the risk of STIs, a finding consistent with previous research [20]. However, the fact that less than half of the participants made this connection highlights a concerning gap in awareness about one of the best-established risk factors for STIs. Sixty-three point eight percent (63.8%) of participants were unaware of the complications associated with sexually transmitted infections (STIs), a notably higher rate than the 50% reported by Subbarao et al. [18].

Only 44.3% of participants had a positive attitude toward STIs, which is considerably lower than the 70.1% reported by Dorji et al. [8]. This lower percentage reflects a persistent stigma surrounding STIs, which may discourage individuals from seeking medical care or disclosing their condition to partners. Interestingly, while 48.6% of participants expressed concern about unprotected sex leading to unwanted pregnancy, a higher percentage (53.4%) were more worried about contracting an STI. This suggests that while pregnancy remains a concern, there is growing awareness about the risks of unprotected sex in transmitting infections. Fifty-five point four percent (55.4%) of participants acknowledged that untreated STIs could lead to death, which is encouraging. This shows that there is a relatively high level of awareness regarding the severe consequences of STIs if left untreated. Only 53% of participants believed that sex education should be implemented at the school level, a significantly lower figure compared to the 93.5% reported by Kumar et al. [21]. Advocacy for comprehensive and age-appropriate sex education programs is critical to shifting attitudes and improving sexual health outcomes in this population. Only 23.1% of participants felt that individuals with STIs should be isolated and avoided, a much lower percentage than the 42% reported by Subbarao et al. [18]. Continued efforts to destigmatize STIs, promote treatment, and advocate for empathy toward those affected are crucial in fostering a supportive healthcare environment and improving public health outcomes.

Strengths of the study

This study's cross-sectional design allows for a broad snapshot of the patient population attending the Venereology OPD. Data analysis was conducted using advanced statistical tools, ensuring robust and reliable results. The study was conducted in accordance with ethical guidelines, with informed consent obtained from all participants.

Limitations of the study

The study was conducted among patients attending the Venereology OPD; therefore, it is unclear whether the findings can be generalized because of the differences in health-seeking behaviors. A cross-sectional study may be prone to a recall bias. Due to the cross-sectional design, causal relationships between variables cannot be inferred. A social desirability bias can lead to an underestimation of high-risk sexual activity. The reliance on self-reported data may introduce a response bias, as participants may underreport stigmatized behaviors or experiences. The study was conducted over a limited period, which could influence the results.

## Conclusions

In conclusion, this study highlights critical gaps in sexual health knowledge, contraceptive use, and attitudes toward STIs among patients at GMCH, Thiruvallur. Despite some encouraging trends, such as lower rates of high-risk behaviors and a relatively higher rate of STI screening, the findings point to a significant need for targeted education and interventions. Low awareness of STI complications, limited contraceptive use, and persistent stigma suggest the importance of improving access to sex education, healthcare services, and STI prevention measures, particularly in rural areas. We must scale up community-based education, strengthen contraceptive supply chains, and promote inclusive STI care, particularly for LGBTQIA+ communities. These efforts will directly contribute to achieving SDG 3, improving overall health outcomes, and reducing health disparities. Collaboration between governments, NGOs, healthcare providers, and local communities is crucial for success. Investments in education, infrastructure, and inclusivity will empower marginalized populations and foster sustainable development. Addressing these challenges can contribute to better sexual health outcomes and reduce the risk of STI transmission.

## Figures and Tables

**Figure 5:**
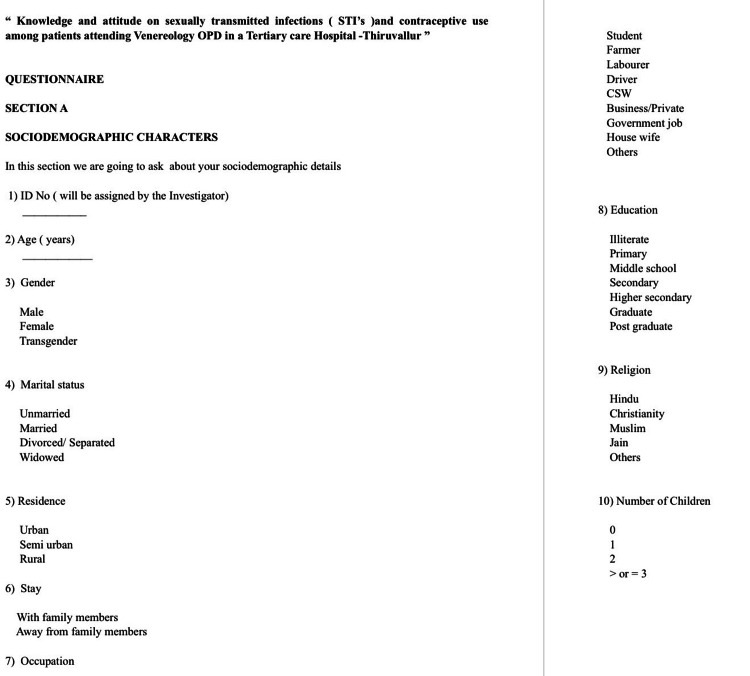
Sociodemographic characters

**Figure 6:**
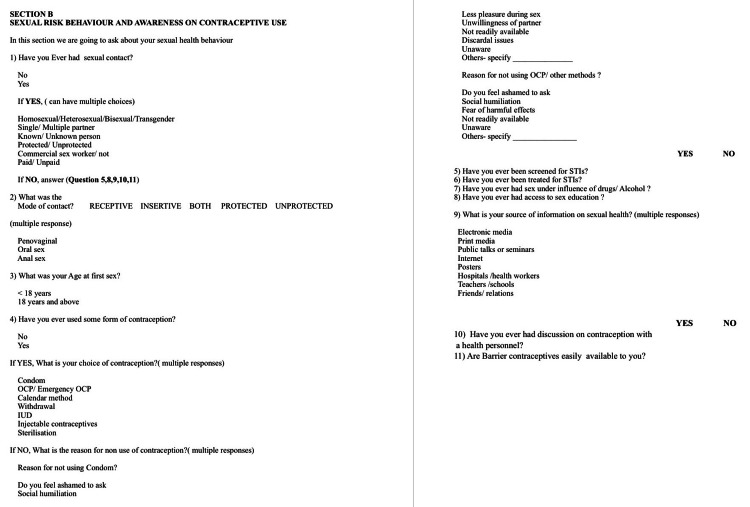
Sexual risk behavior and awareness of contraceptive use

**Figure 7:**
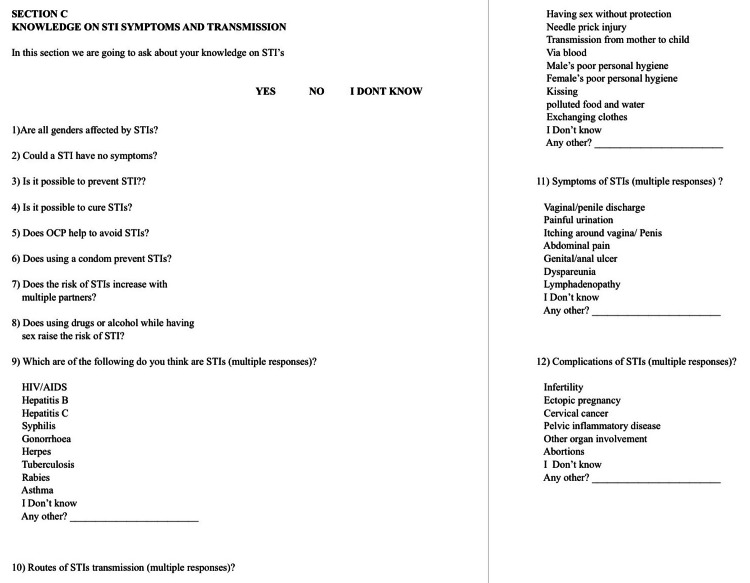
Knowledge of STI symptoms and transmission STI: sexually transmitted infection

**Figure 8:**
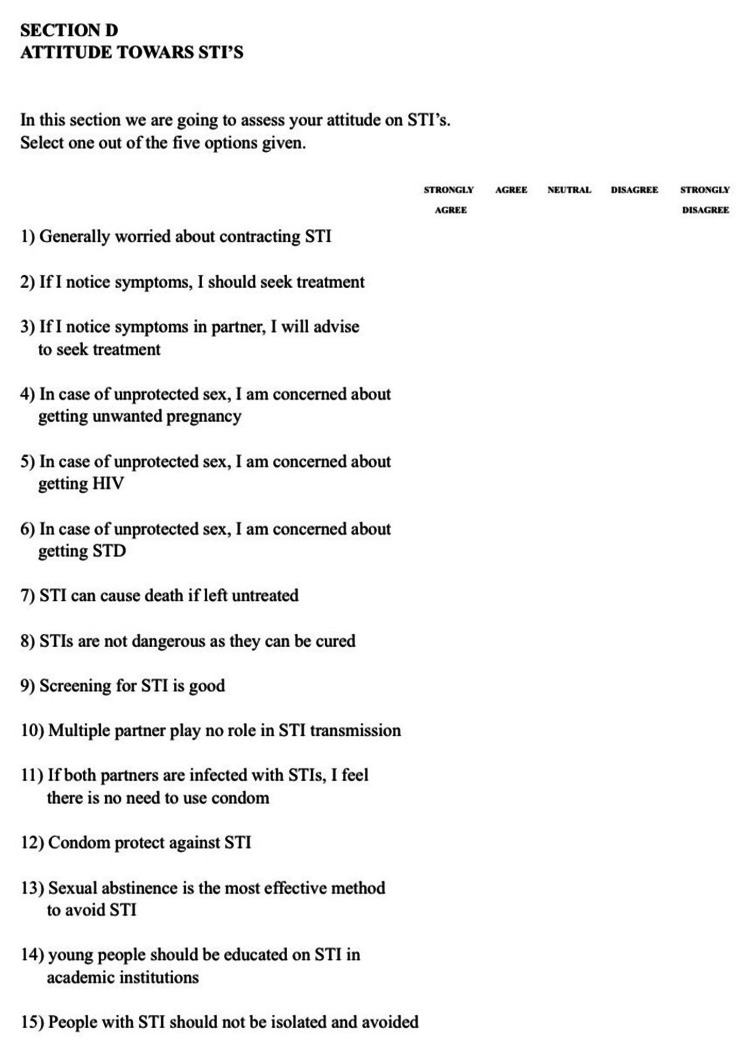
Attitude toward STI STI: sexually transmitted infection
